# Surviving transition: A qualitative case study on how families adapt as their youth with medical complexity transitions from child to adult systems of care

**DOI:** 10.1016/j.hctj.2023.100035

**Published:** 2023-12-16

**Authors:** Lin Li, Nancy Carter, Jan Willem Gorter, Linda Till, Marcy White, Patricia H. Strachan

**Affiliations:** a School of Nursing, McMaster University, Hamilton, ON, Canada; b Canchild Centre for Childhood Disability Research & Department of Pediatrics, McMaster University, Hamilton, ON, Canada; c Department of Rehabilitation, Physical Therapy Science and Sports, UMC Utrecht Brain Center, University Medical Center Utrecht, Utrecht, the Netherlands; d Parent Research Partner

**Keywords:** Transition to adulthood, Transition to adult health care, Youth, Adolescents, Medical complexity, Complex care

## Abstract

**Background:**

A growing population of youth with medical complexity (YMC) are entering adult health care, education, and social systems in which their needs have been largely neglected. To better support YMC and their families, an understanding of how they manage the challenges of transitioning to adult services is needed. The aim of this study was to examine how families of YMC adapt to challenges and opportunities posed by the youth’s transition to adulthood and transfer to adult services.

**Methods:**

In partnership with two parent co-researchers and underpinned by complex adaptive systems and the Life Course Health Development framework, a qualitative explanatory case study was conducted. Seventeen participants from 11 families of YMC (aged 16–30) living in Ontario were recruited. Data from 21 semi-structured interviews were analyzed using reflexive thematic analysis and further refined through theory-driven analysis. Supplementary documents shared by participants were analyzed using directed content analysis.

**Findings:**

Three overarching themes were generated. “Imagining, pursuing, and building a good future” describes families’ priorities and visions for the youth’s life as an adult. “Perils and obstacles of an imposed transition” examines challenges that families face in their pursuit of a good future. Lastly, “surviving the transition” describes how families are forced to advocate, make sacrifices, and persist in their efforts to adapt to transition.

**Conclusions:**

Pediatric providers should offer anticipatory guidance, partner with families in advocacy, and provide psychological support during transition. Education for adult and primary care providers should focus on developing professional competencies in the safe care of YMC, building capacity through clinical exposure, and creating culturally safe environments. Most importantly, YMC and their families need a model of care that can provide integrated, holistic, multidisciplinary care management across the lifespan.

## Highlights

• Families prioritize health, safety, participation and autonomy for the YMC’s future.
• Families fear their YMC’s health will suffer due to lack of trained adult providers.
• The culture of the adult system leaves families feeling unsafe and unwelcome.
• Caregivers weather the effects of prolonged, intense caregiving/unpredictability.
• Families adapt to transition through survival, sacrifice, persistence and reframing.

## 1 Background

Youth with medical complexity (YMC) are a growing population of young people with multiple chronic conditions, functional limitations, and extensive caregiving and service needs.^1^ Their care is often complicated by a myriad of medication and therapy regimens, frequent hospitalizations, and reliance on medical technologies for vital functions.^2^, ^3^ Their care also involves multiple medical specialists, primary care providers (PCPs), nurses, rehabilitation professionals, and other health, education, and social service providers.^4^ To meet their extensive health needs, team-based complex care programs have been widely implemented across Canada and the United States. These programs, which provide comprehensive and coordinated care, have shown promising results related to patient, family, and system outcomes.^5^, ^6^, ^7^, ^8^

While there has been heightened interest in improving the care of YMC in the pediatric world, their needs have been largely neglected once they “age out” of the child health care system.^9^, ^10^ For these youth and their families, the transition to adult care (i.e., health care transition) has often been compared to “falling off a cliff.”^10^ This experience is due, in part, to a combination of: the lack of equivalent adult services to replace essential pediatric services (e.g., multidisciplinary complex care programs), the termination of long-standing relationships with pediatric providers, the scarcity of knowledgeable adult providers, and the difficulty families face in navigating a completely new adult-oriented system.^9^, ^10^, ^11^

Efforts to improve health care transition have primarily focused on assessing transition readiness and cultivating self-management skills.^12^ However, these interventions have limited benefits for YMC, who often have neurologic impairment and/or developmental disabilities that limit their capacity for independence and self-management.^1^ Additionally, research on health care transition often focuses on disease-specific, specialist-to-specialist transfers,^13^ failing to consider the compounding effect of numerous health care transfers that YMC inevitably go through. It is widely recognized that transitions of care are especially challenging for this population.^14^, ^15^

Health care transition is embedded in the broader transition to adulthood, which in this study, is conceptualized as “a developmental process in which youth and their families experience physical, psychological, social, and cultural shifts associated with becoming an adult.”^16^ Further complicating health care transition and the transition to adulthood are co-occurring service transitions within non-health systems. This added layer increases the risk for service fragmentation, as YMC regularly interface with multiple systems of care (health care, social, education).^1^ Ultimately, families are left to weather the effects of service gaps and fragmentation within and across systems of care.^17^ Under these conditions, caregivers often assume the role of their child’s 24/7 care coordinator, which can negatively impact their physical and mental health, along with their family and personal lives.^17^, ^18^, ^19^

To better support these families, it is critical to understand how they manage these transitions, including specific challenges they face and how they confront these challenges. Understanding the complexity of transition also requires consideration of the wider context, including how various service transfers intersect to affect the lives of YMC and their families. Therefore, the aim of this study was to understand how families of YMC adapt to challenges and opportunities posed by the youth’s transition to adulthood and transfer to adult health care, education, and social services.

### 1.1 Health system context

In the province of Ontario, Canada, health care is federally/provincially funded and provincially administered. Single-payer, universal health insurance covers medically necessary hospital, diagnostic, and physician services.^20^ Additional health care services and resources (e.g., medications, medical and assistive equipment, rehabilitation services, respite, and more) are paid for through a mix of private insurance, government programs, and out-of-pocket expenses.^21^ In 2015, a province-wide strategy was launched to expand access to comprehensive care and coordination for YMC.^21^ However, at age 18, the provincial government mandates that all youth must transfer from the pediatric to adult health care system,^2^ where no equivalent programs exist.

## 2 Theory

Complex adaptive systems^22^ and the Life Course Health Development (LCHD)^23^ framework provided the theoretical basis for this study. Complex adaptive systems have emergent properties that are not observed in simpler states, meaning an understanding of the system cannot be fully achieved from studying its individual parts.^24^ Using this theoretical perspective, we examined transition through a holistic lens that considers how the youth and family’s developmental and life transitions intersect with service transfers as the youth reaches adult age. Underpinned by complex adaptive systems, the LCHD framework aims to explain how health develops over the life course.^23^ Within this framework, an individual’s adaptive responses to environmental experiences and exposures influence their health over time, particularly during time-sensitive periods, such as the transition to adulthood.

**Fig. 1:**
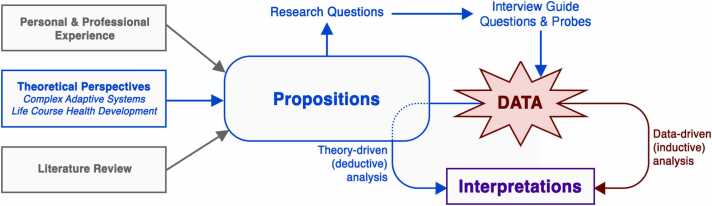
Integration of theoretical perspectives and propositions in study procedures.

These theoretical perspectives were drawn on to define the case, develop theoretical propositions (i.e., hypothetical statements about the expected findings of the study), and enrich the interpretation of findings. Through the propositions, these theories were also indirectly operationalized in the tools and methods for data collection and analysis (Fig. 1). Two theoretical propositions guided this study: (1) families of YMC will have the capacity and motivation to adapt to challenges and opportunities posed by transition and will do so to achieve their goals; and (2) each family’s transition experiences will encompass multiple interrelated transfers within the health care, social, and education sectors.

## 3 Methods

A detailed study protocol has previously been published.^16^ Only a brief summary of the methods and any modifications to the published protocol will be described in this article.

### 3.1 Design

We used a qualitative explanatory case study design,^25^ which allowed for an in-depth, real-world exploration of family adaptation (the “case”) in the context of the YMC’s transition to adulthood and transfer to adult services. Two parent co-researchers with lived experience contributed to study design, grant application, recruitment, data analysis, and knowledge translation.

### 3.2 Sampling and recruitment

Purposive sampling strategies were used. Criterion sampling identified eligible participants that: 1) were a YMC or their family member; 2) were aged 16 years or older; 3) were living in Ontario, Canada; 4) spoke English; and 5) had experience with the youth’s transition to adulthood or transfer to adult services. In this study, YMC were defined as individuals who: 1) were aged 16–30 years; 2) had a chronic health condition; 3) required extensive support from caregivers or medical technology for vital functions or activities of daily living; and 4) required care from five or more health care providers while in the pediatric system.

Maximum variation sampling identified participants who: 1) lived in both rural and urban settings; 2) were both pre- and post-health care transition; and 3) had both positive and negative experiences with transition. A sample size of 10–15 families was sought, with 1–3 participants from each family. This number was expected to be large enough to yield findings that would be supported by multiple perspectives,^25^ while also being small enough that an in-depth exploration of experiences was feasible. Participants were recruited from the community, through a variety of electronic and social media. Those interested contacted the primary author, who then screened for eligibility and explained the study details.

### 3.3 Data sources

Data sources included semi-structured interviews, documents, field notes, and memos. During the interviews, participants were asked to share documents, tools, and other resources that they found helpful in their transition experiences. All interviews were conducted by the primary author using Zoom videoconferencing software, recorded, and transcribed verbatim. The primary author also documented field notes to add context to the interviews and wrote memos to journal the development of ideas.

### 3.4 Data analysis

Data were analyzed by LL and PS using NVivo 12. Reflexive thematic analysis, a theoretically and epistemologically flexible method, was used as a data-driven approach to analysis.^26^ Semantic themes that foreground the experiences and realities of participants were developed inductively into an overarching storyline (i.e., explanation building).^25^ Subsequently, by examining each family’s experiences against the original theoretical propositions, theory-driven (deductive) analysis further validated and refined the initial themes. Tools and resources shared by participants were analyzed using directed content analysis and were used to validate and supplement the interview findings.^27^

Following analysis, the full research team was involved in peer auditing and interpretation. As a team that includes members with and without lived and/or clinical experience, we were cognizant of the influence and value of our positionality on data collection and analysis, and interpretation of findings. Lastly, a lay summary of the findings was emailed to participants, and their feedback was used to further refine the findings (i.e., member checking).

### 3.5 Ethical considerations

This study received ethical approval from the Hamilton Integrated Research Ethics Board under project number 11184. Informed consent was obtained electronically and was reaffirmed verbally at the start of each interview.

## 4 Findings

There were 17 participants in this study representing 11 families of YMC. In all families, the mother (n = 11) was the primary caregiver, and in five families, the mother was the sole participant. In the remaining six families, participants included: a mother and a YMC (n = 2), a mother and father (n = 2), and a mother and sibling (n = 2). While all youth met eligibility criteria for medical complexity (see Sampling), they had a myriad of medical problems affecting all body systems. Some common diagnoses within this group included cerebral palsy, developmental delay, autism, seizure disorder, gastroesophageal reflux disease, and chronic pain. These youth also had a variety of rare genetic, neurodegenerative, and/or life-limiting conditions. See Table 1 for youth and family characteristics.

**Table 1:** Youth and family characteristics.

Characteristic	*N*
*YMC age (range = 16–28 years; n = 11)*	
Under 18 years (pre-health care transfer)	6
18 years and over (post-health care transfer)	5
*YMC gender (n = 11)*	
Female	7
Male	4
*Participant gender (n = 17)*	
Female	13
Male	4
*Family structure (n = 11)*	
Parents are married	8
Parents are single, widowed, or divorced	3
Youth has siblings	8
Youth is the only child	3
*Residential area (n = 11)*	
Urban	7
Rural	4
*Annual household income in Canadian dollars (n = 11)*	
Less than $25,000	3
$75,000–99,999	2
$100,000–149,999	2
$150,000–200,000	1
Prefer not to answer	3
*Caregiver age (n = 13)*	
36–45 years	2
46–55 years	9
56–65 years	2
*Caregiver education (n = 13)*	
College diploma/certificate	5
Bachelor’s degree	4
Graduate degree	3
Prefer not to answer	1

Twenty-one individual interviews (including 4 repeat interviews) were completed, each lasting 30‐100 minutes. Three major themes were generated in response to the overarching research question of “how do families of YMC adapt to the youth’s transition to adulthood?” The first theme of “imagining, pursuing, and building a good future” describes families’ visions, goals, and priorities for the youth’s future as an adult. The second theme of “perils and obstacles of an imposed transition” examines key experiences and exposures that pose adaptive challenges to families’ pursuit of a good future. The third theme of “surviving the transition” embodies families’ experiences of being forced to adapt to imposed service transitions and living with the resulting inequities. Additional participant quotes that support the following narrative can be found in Table 2.

**Table 2:** Themes and additional supporting quotes.

Theme *Subtheme*	Supporting Quotes
**Imagining, Pursuing, and Building a Good Future**	“So, for us, number one is safety and of course, health, right?” (Mother B) “I knew that the school years were going to be the best years because we have fully funded education through the government. There's people. He's going to make these relationships with these [educational assistants] and these students in his class. And it's going to be wonderful. But when it's over, it's over. And I know then it's up to us.” (Mother E) “If she gets to the point where she’d have her own apartment and she has friends she can talk to…a place she can call home, where she’s proud of all her stuff—that’s what I would ideally envision for her.” (Father I) “Until my dying day, she will live right here beside me.” (Mother G)
**Perils and Obstacles of an Imposed Transition**	
*Rupture from the Familiar*	“These people have invested care into [Youth I] and like kids for, well, like in her case, 18 years.” (Father I) “We just left [Children’s Hospital B]. So we went from a team of about 12 to nothing.” (Mother G) “Her pediatric cardiac specialist put a letter of reference to a new cardiologist, but the cardiologist refused her. Or the pediatric neurologist put in references to new adult neurologists, and the adult neurologist refused her.” (Mother J) “So we're going to flip over to our family doctor, which quite honestly, is not going to be specialized at all for [Youth K]'s needs. But he will be there to do the simple things, like renewing the prescriptions and that sort of thing.” (Mother K)
*Exile to a Perilous New World*	“Instead of going to the one place like [Children's Hospital] where everybody knows you and you're used to the system, you're used to the whole layout of the hospital, and now we're going to be just going all over the place.” (Mother A) “When a child is in the hospital, you'll have so many different specialists involved. And if that specialist works at the hospital and they know that one of their patients is in, they'll often pop in, even if it's not related to that illness. At least that was my experience.” (Mother F) “[Allied health professionals] would have their set schedule for going to see [Youth F] at school, like PT, OT, speech—and in adult world it's only when I request.” (Mother F) “It’s exhausting to have to go through all that again and to have to prove yourself, that you actually know better. And if you’re talking to them, that means you’ve already done the first several steps of troubleshooting. This is real.” (Father A)
*“A Bureaucratic Nightmare”*	“Getting his own place with his own workers and everything—that was a mental, psychological and bureaucratic nightmare… Watching my parents kind of try to jump through loopholes with the government.” (Sibling C) “We have a six hour interview that we have to pay a few hundred dollars for to prove that she's not miraculously cured at the age of 18.” (Father A) “I think the whole process of how the transition happens is not clear. Like I did not know. I was clueless.” (Mother D)
*Weathering the Effects of Prolonged, Intense Caregiving*	“My son also has a sleep disorder. So it's been like having a newborn for 27 years. So it was never like we could ever recoup or catch up.” (Mother C) “Their needs actually increase because they often deteriorate as they age. They're also physically harder to manage and the parents are also aging and many are going through health issues themselves.” (Mother F) “It was much easier finding help for our daughter when she was five years old and tiny compared to now when she is much bigger.” (Mother A) “Because we were in crisis, we literally reached out to the ministry and said, 'We're done…We're going into crisis. We cannot spread ourselves any thinner.’” (Mother C)
*Living with Unpredictability, Enduring Profound Emotions*	“If we're up to me, nothing should change, because obviously when there's any, like, medical complexities, developmental delays, mental health, any of these situations, then you need just as much help. It doesn't matter if the child turns 18.” (Mother B) “Yeah, well, it's a big change, right? And our best days is when there are no changes.” (Father A) “Why can't we just leave well enough alone and leave her at [Children's Hospital]?” (Mother G) “I'm grateful for every day with her.” (Father A)
**Surviving the Transition**	
*Transition Work and Advocacy*	“I'm constantly following up with everybody to find out, has this referral been made? Who has this referral been made to? I haven't heard from anyone. Is there a number that I can contact? Do you have any more information? Do you know when I'm going to see this person? What should I expect? What happens if I don't hear from anyone?” (Mother H) “I've been thinking creatively about what we can do with what we have.” (Mother E) “I am involved with a local group through one of the churches in town, and we have established a group for adults, 21 and over, who have mental and physical disabilities—to kind of come together once a week in a completely social setting…Our goal was to have it a longer period of time, build the capacity up, because they do recognize that there's a big gap, that there's nothing for adults.” (Mother K) “And I called the ministry and said, 'Something's got to give, or I'm going to bring him to a hospital, and I'm going to walk away,' which…I would never be able to do. But in that moment, that's what I felt like.” (Mother C)
*Family Sacrifices and Role Changes*	“But it's too bad there wasn't more support so that we don't have to do it all, that WE could retire, that WE could travel, that WE could do things.” (Mother E) “I just remember the sense of profoundness in the air, like, 'Oh, my goodness, my brother is going to get his independence finally. And my parents, for the first time in their parenting and adult life, are going to get their independence back to an extent as well.” (Sibling C). “But it is so tricky because we don't know yet what the personalities of our other two kids will be. Maybe they won't be able to handle that. Maybe they will be in situations where that's just not going to work. So that's the scary part” (Mother E).
*Persisting Through Hardship*	“Who will take care of her when I’m gone, right? But I can’t live like that. Every day is a blessing” (Mother G). “I felt like I was blazing trails and that nobody before me had done exactly what I was doing. But it turns out that there were families before me that were doing the exact same thing and fighting for the exact same thing. And they thought that they'd made an impact on making changes, but in fact they hadn't.” (Mother F) “Any changes that have supported our goals for the future are because we have put huge work into it and tried to dress it up with a multilayer costume.” (Mother J)

### 4.1 Imagining, pursuing, and building a good future

Families described a good life in adulthood as the youth living a happy, full life. At a minimum, this meant the youth having their needs met and being socially engaged and included in their community. Families’ hopes and fears for the youth’s future were guided by three common principles: health and safety, social life and participation, and independence and autonomy.

Due to their profound disabilities, medical complexity, and/or health instability, protecting the YMC’s health and safety trumped all other concerns. Families described being forced to adjust their goals from thriving—or as Mother J put it, “*having a full and meaningful, engaged life*”—to surviving the experiences of transition and the adult care system. For example, Mother A shared, *“I would rather my child dies than risk her being abused in a group home somewhere.”*

Another major priority for caregivers was finding ways to continue to keep their adult children engaged within their family’s means. “Aging out” of the school system meant the loss of full-day programming that offered the youth social interaction and participation in engaging activities. Many YMC do not have the ability or required support to pursue post-secondary education or work, and limited day programs often excluded youth with higher needs or required costly attendant care. As a result, these families are forced to assume the burden of replacing essential supports and social engagement that were previously universally accessible through school.

Independence and autonomy, for both the YMC and their caregivers, was another common hope and concern. Some caregivers imagined living and caring for the youth indefinitely, while others hoped to lead more separate lives from their children. These goals for independence and autonomy varied based on each family’s unique circumstances, capacities, and access to dependable alternate care arrangements.

### 4.2 Perils and obstacles of an imposed transition

In striving to achieve their visions for the future, families experienced physical, emotional, relational, and structural processes that made the transition difficult. These processes are described by the following five subthemes: (1) rupture from the familiar; (2) exile to a perilous new world; (3) “a bureaucratic nightmare”; (4) weathering the effects of prolonged, intense caregiving; and (5) living with unpredictability, enduring profound emotions.

#### 4.2.1 Rupture from the familiar

When YMC age out of the pediatric health care system, families must leave trusted health care providers and familiar services and settings. Many families described having strong relationships with their pediatric providers, some of which had cared for the youth and family for the entirety of the youth’s life. Caregivers worried that their new providers would not know them on a deep, personal level, and that this would compromise the youth’s health and safety.

While some families described their pediatric providers as strong advocates who helped make the transition easier, others felt abandoned by both pediatric and adult providers during transition. Families felt abandoned when pediatric providers ended their services abruptly, or when adult providers refused referrals to take over care. Mother G described her transaction-like experience with transferring to a new provider: *“There’s nothing you can do. They just say, ‘OK, this is your last appointment. Here’s your new specialist. Good luck. See you later.’”*

For youth that would transfer from a large multidisciplinary care team to a lone PCP, their families worried that the youth’s highly complex care needs would be beyond the PCP’s resources, knowledge, and experience. These concerns were validated by experiences of families who had already transitioned from a comprehensive care program to a PCP, as Mother F shared, *“Our family doctor didn't want to take [Youth] on because she was too complex… Our family doctor now is really limited… She can't even order blood work on [Youth] and [Youth] has a port-a-cath.”*

#### 4.2.2 Exile to a perilous new world

YMC are exiled from the pediatric system and dropped into an adult system that was not designed for them. In this perilous new world, families experience drastic shifts—in the structure, delivery, and culture of health care—that makes them feel unsafe and unwelcome. Families compared receiving multidisciplinary pediatric care in centralized locations to adult care that was often delivered by lone providers in many different locations. Families also reported that their pediatric providers would check in proactively with the youth, even when they were well. On the other hand, adult care was perceived to be offered in reaction to active health issues. Due to this shift to a more reactive care model, families had to be more vigilant in monitoring and managing the youth’s health. Furthermore, families reported that adult services tended to be less holistic and more problem-focused, as Mother J described her experience with adult social work: *“They want to be able to fix the issue in two visits or three visits. Complex disabilities and issues like [Youth J]'s don't fix in two or three half-hour visits.”* These changes led to a disconnect between the kind of care families needed and expected and the care that was received and experienced.

Furthermore, many families felt a tangible shift in health care culture, from a family-centered philosophy of care to a culture in which caregivers are less visible. Caregivers worried that they would lose the respect and authority they garnered within the pediatric system—that they would have to “prove themselves” all over again as knowledgeable experts of their youth’s medical needs. This cultural shift was most shocking to families who had experienced adult hospital admissions. Families perceived adult hospitals to be “scary” and “unsafe,” due to both the physical environment (e.g., lack of appropriately sized equipment) and the cultural environment (e.g., perceived “ableist” attitudes of health care staff). Mother J observed:

*The adult attention and cares for complex individuals like her are very, very different than what you get in [the pediatric system]. It is like you fall off the face of the earth and you have to prove that she continues to be human and continues to be worthy of life-sustaining treatment.*

#### 4.2.3 “A bureaucratic nightmare”

Families were also frustrated by the bureaucratic processes of transition, which involved lengthy applications, assessments, and legal procedures to determine the youth’s eligibility for adult services. Sibling C described the process as “*a mental, psychological, and bureaucratic nightmare.”* Families felt that they should not have to prove that the youth’s care needs would remain the same from one day to the next. Some families also described having to pay out of pocket to obtain guardianship or psychoeducational assessments that were required for the youth to access government-funded adult services.

Furthermore, different rules exist within the health care, social, and education systems that govern when and how youth can access services within specific regions. These different rules increased the complexity of transition, especially as many families shared that transition was made more difficult by not knowing the steps and prerequisites for securing adult services. Mother D commented that “*there shouldn’t be a hundred different things that we have to do because we’re dealing with different ministries.*”

#### 4.2.4 Weathering the effects of prolonged, intense caregiving

Further exacerbating the challenges faced during transition was the timing in which these changes intersect in the broader context of the youth’s and family’s lives. YMC have extensive care needs, and transition occurs at a time when caregivers are weathering the effects of almost two decades of intense caregiving. Furthermore, YMC often have increasing care needs as they grow bigger or as their health declines, making it more difficult to find paid caregivers and further compounding the burden placed on families.

Caregivers feared their own future aging and possible declining health would limit their capacity to provide physical care and financial support for their adult children. Mother C stated, *“He gets bigger, you get older. You're constantly financially and emotionally drained.”* Other caregiving and family demands also made it harder for caregivers to cope effectively with the demands of transition, as Mother I explained, *“You’re kind of exhausted because this isn't my sole thing that I do.”* The heavy burden of the youth’s ongoing—and often increased—care needs, coupled with caregivers’ possible declining health, put some families in crisis situations where they were unable to cope with the demands of daily life, let alone dedicate time and energy to managing transition.

#### 4.2.5 Living with unpredictability, enduring profound emotions

For the caregivers in this study, transition was fraught with grief from multiple sources, such as ongoing uncertainty of the youth’s health status and lifespan. Some caregivers described the transition to adulthood as “*bittersweet*” and “*unexpected territory*,” as they had not expected their child to survive into adulthood. Due to the fragile health of YMC, these families continue to weather the compounding and traumatic effects of years of living with uncertainty.

The fragile and unpredictable nature of the youth’s health also fueled caregivers’ apprehension toward changes brought on by transition. These imposed changes did not make sense to families of YMC who were not expected to survive long into adulthood or who would remain like a child physically or developmentally. Mother A observed, *“There's not a whole lot that's changing with her, and she is like a child, but her age, because she's 18, suddenly she goes into adult services. And it just doesn't make a lot of sense for me.”* Families valued consistency and continuity of care, which can offer a sense of security to help ease the vast uncertainty that permeates their day-to-day lives.

Another major source of grief for caregivers stemmed from considering who would take over care should their child outlive them. Mother I reflected, *“I didn't realize how from an emotional perspective, I wasn't ready for this transition… I think it's because, as parents, we do think about the end of our life.”* Even families who felt well-supported experienced unpredictability, lack of agency, and fear that future political changes would result in essential services and supports being stripped away from them, as Mother C described:

*My fear is that after everything I've done in my life for him, that when I am no longer able to be his voice, that he's going to get dumped back into the system. Because every year when the funding gets renewed, I have to get ready… to plead my case and basically beg to continue on for another year.*

Despite the overwhelming negative emotions and experiences associated with transition, some families described positive outcomes of transition. Families felt grateful to continue spending time with the youth, and they were excited to see the youth develop and live in new ways as an adult. Sibling D reflected, *“She's really kind of grown up in the past years and really started developing into someone, into her own self.”* However, positive experiences related to service transitions were often isolated to local pilot programs or unique circumstances engineered through a family’s monumental advocacy efforts.

### 4.3 Surviving the transition

The final theme of “surviving the transition” describes the essence of how families of YMC adapt to the youth’s transition to adulthood. Families survived this imposed transition by putting in tremendous amounts of work and advocacy, making significant sacrifices and role changes, and persisting despite the hardships they faced.

#### 4.3.1 Transition work and advocacy

The day-to-day “work” of navigating transition mostly fell to the youth’s primary caregiver, which in this study, were all mothers. These mothers put in enormous efforts to prepare for and survive the transition experience. Transition work involved discovering the ins and outs of the system, stumbling over the rules, and figuring things out on one’s own. Mother K commented, “*But nobody really walked me through the process. It was me kind of learning it on my own. You know, what did I need to do and who did I need to contact?”* Caregivers had to be resourceful, organized, and persistent, and they had to be strong advocates for services, programs, and system change.

Limited programming and options meant that families often had to think creatively to “make it work” with the limited resources that were accessible to them. Families imagined and worked toward building new options for a brighter future for their children. In some cases, families described being driven out of desperation to enact “extreme” survival measures to push against the bounds of the system. After being put through the gauntlet of transition, families were forced to live with the resulting outcomes and inequities, determined partly by serendipity and partly by their mastery of the system.

#### 4.3.2 Family sacrifices and role changes

Transition affected the whole family, with family members being forced to make significant sacrifices and role changes. YMC and their families were frequently forced to alter or sacrifice their goals, which were often not supported by the bounds of the current health care, social, and education systems. Some caregivers desired to have a life and identity outside of caregiving, yet they were forced to sacrifice their careers or reduce their work hours to meet the increased demands of caring for their adult child. Mother J shared, *“I am now home with her full time because I can't afford to pay for the level of care that she needs in the community because of her complexity.”*

For families with multiple children, parents spoke of their hope that the youth’s siblings would eventually take over care. Father I stressed the importance and longevity of the sibling relationship: *“The siblings are normally going to be the people who have the relationship the longest with each other*—*longer than parents, longer than your doctor, longer than a caregiver, longer than your friend, by a mile.”* However, parents also feared how caregiving would impact the sibling’s life, and they expressed uncertainty about relying on the youth’s siblings to become caregivers. Although the sibling participants in this study wanted to maintain a close relationship with the youth, neither spoke of their future roles as caregivers.

#### 4.3.3 Persisting through hardship

Families also persisted through hardship by coping psychologically and reframing their lives. Some families coped with the stressors of transition by focusing on the present and not dwelling on the uncertainties of the future. Participants spoke of the importance of being resilient, having a positive outlook, and maintaining hope for the future, as Mother H describes: “*Over the years, we've had to change and adapt and keep moving forward. We've always been resilient and…we know that we've gotten this far, that we will do whatever it takes to keep going.”*

Families felt both encouraged and disenfranchised by the ever-changing social, political, and economic landscape. Families who were many years into transition revealed that they still did not feel situated within the adult system, as exemplified by Mother F: *“We’ve been 10 years now in that transition and I can’t say that I’m fully accustomed to it.”* Although some families—through persistence and strong advocacy—were able to set up care or housing options that worked for them, these successes appeared isolated. Families were dismayed at the lack of long-term systemic change for issues that have persisted since before their transition journeys began. This continued inertia leaves families feeling as if their children are “forgotten” in the current system. Mother D observed:

*How long has this been going on and nothing has changed? These people are the forgotten people of Canada. And what has to happen for people to start paying attention that these people, once they turn 18, are tossed aside and forgotten?*

These families confront fear and uncertainty, endure the obstacles and hardships of transition, and persevere within a system in which they feel invisible. Despite their immense advocacy efforts and sacrifices, they are often forced to reframe their lives to cope with the inequities worsened by transition.

## 5 Discussion

This qualitative case study examined how families of YMC adapt to the youth’s transition to adulthood and transfer to adult services. While previous research has explored youth and family transition experiences among similar populations—such as youth with life-limiting conditions,^9^, ^28^, ^29^ neurological disorders,^30^, ^31^ intellectual disabilities,^32^ and mechanical ventilation needs^33^—this study is among the first to examine these experiences for families of YMC,^34^ and to our knowledge, the first in Canada. Furthermore, by holistically examining transition for YMC beyond the health care system, we offer novel and timely insights that align with calls to consider the bigger picture of transition across multiple systems of care.^9^, ^35^, ^36^, ^37^ The engagement of parent co-researchers also contributed to interpretations that increase the relevance of the findings and implications for families.

### 5.1 Addressing initial theoretical propositions

By comparing our data to the initial theoretical propositions, we were able to expand our understanding of how these families adapt to transition. The first proposition was that “families of YMC will have the capacity and motivation to adapt to challenges and opportunities posed by transition and will do so to achieve their goals.” We found that the transition experience is often fraught with extreme fragility, and families’ capacities for adaptation are linked to the dynamic unpredictable conditions that they live with. In preparing families for transition, providers need to acknowledge the overwhelming uncertainty that families face by offering psychological support, anticipatory guidance, and flexible trauma-informed approaches to care that can account for unpredictable conditions and health trajectories.

Furthermore, we originally conceptualized adaptation to be a goal-oriented process. However, we found that for some families of YMC, adaptation often devolved into a survival-oriented process. These families were so focused on coping with their present unpredictable conditions that they had limited capacities to pursue their visions for the future. Even when the challenges of transition overwhelmed their capacities to cope, their motivation to survive forced them to stretch beyond sustainable and healthy capacities for hardship. This finding reveals the overreliance of current health and social systems on the invisible work of families and calls to question the sustainability of such a system.

The second theoretical proposition stated that “each family’s transition experiences will encompass multiple interrelated transfers within the health care, social, and education sectors.” We discovered that in addition to the number of interrelated service transfers, the timing in which they occur in the youth and family’s lifecourse is also critical. This stressful transition occurs at a time when caregivers are exhausted from enduring two decades of intense caregiving, potentially experiencing their own aging-related health issues, and taking on additional caregiving responsibilities (e.g., for their parents). Interventions and policies to support transition need to consider the timing of imposed service transfers and provide support for common challenges affecting family caregivers at this point in their lifecourse.

### 5.2 Implications for practice, policy, and education

#### 5.2.1 Health care provider training

Families in this study reported difficulties in connecting with competent and willing adult providers. These challenges have been well documented in other studies.^34^, ^38^, ^39^ To improve the quality of care that these families receive, health care provider training should focus on competency in caring for YMC and the creation of culturally safe environments.

##### 5.2.1.1 Competency

Other studies have reported that there is a perception among health care providers and families that general practitioners, adult providers, and non-pediatric nurses lacked the skills and knowledge to manage the care of this population.^34^, ^40^ Yet, YMC and their families interface with all practice settings, from pediatric to adult, generalist to specialist, and primary to tertiary. Thus, it is paramount that training in, and exposure to, the care of YMC is incorporated into the education of diverse pediatric, adult, and primary care professionals. Glader and colleagues^41^ have proposed a framework of professional competencies in the care of YMC, which can serve as a foundation for developing clinical training initiatives and equipping health care teams with the necessary expertise to safely care for this population.

PCPs are well positioned to support health care transition, as their scope of practice across the lifespan affords them connections to both pediatric and adult specialists. Yet in Canada, many YMC do not regularly see a PCP, and this lack of exposure renders them invisible and excluded from efforts at primary care reform.^42^ To increase exposure and lay the foundation for capacity building, complex care providers should engage and partner with PCPs in the care of YMC from an early age. Our calls to increase training and exposure to YMC, particularly within primary care settings, echo recent recommendations made by the US-based National Alliance to Advance Adolescent Health, Got Transition, and the Fitzhugh Mullan Institute for Health Workforce Equity on strengthening adult primary care to better support YMC.^43^

Furthermore, lack of competency in the care of YMC, coupled with the limited resources of the adult health care system, has led to some YMC being turned away from adult providers. Unable to find suitable providers, YMC are subjected to delays or gaps in care that can lead to health declines or life-threatening events.^34^ Thus, during all transitions of care, it is of utmost importance that transferring providers ensure that YMC are securely connected to safe and competent care. Pediatric providers should not relinquish their responsibility for care until this requirement can be met in the adult system.

##### 5.2.1.2 Culture

In this study, families shared alarming stories of unsafe care experiences and ableist attitudes within the adult health care system. Roy and colleagues^34^ also reported that families of YMC felt dismissed by adult providers, who were not used to the involvement of families in their patients’ care. This lack of respect for caregivers, coupled with the reactive, episodic care models used in adult health care, means that caregivers are increasingly relied on to manage their youth’s health care needs, while simultaneously being stripped of the authority and respect that their role demands. As one of our parent research partners explains, “families, mostly mothers, must carry the full medical history of their child into every medical setting, and must be educators (often patronizingly responded to) and vigilant protectors of their child to ensure no harm occurs.” To create culturally safe environments within adult care settings, health care provider training should also focus on reducing stigma towards disability and education in developmentally appropriate, family-centered models of care.

#### 5.2.2 A new model of care

A unique challenge that YMC and their families face is the absence of equivalent adult complex care services to transition to. As a result, their primary care is often being transitioned from a large multidisciplinary team to a lone PCP with limited experience and resources to care for YMC.^13^, ^40^, ^42^, ^43^, ^44^ Despite decades of efforts to improve health care transition, there has been little change within the adult system to meet the needs of this population. As a growing population of YMC are reaching the age of transition,^45^ it is increasingly unacceptable that there is no system in place to safely care for young adults with medical complexity.

We need a model of care that can provide comprehensive management across the lifecourse. Advanced practice nurses (APNs), with their broad scope of practice and holistic purview, are well positioned to lead this model of care, especially as APNs already effectively manage the care of YMC within the pediatric system. Furthermore, numerous studies on nurse-led models of care for adults with multimorbidity or complex chronic conditions have shown promising results, such as improvements in care quality, access, clinical outcomes, and emergency service use.^46^, ^47^, ^48^ Most importantly, this approach needs to be integrated and multidisciplinary, with the option of consulting both pediatric and adult specialists based on the patient’s needs and health conditions, regardless of their age.

### 5.3 Limitations

A key limitation of this study was accurately identifying medical complexity. Potential participants were asked to self-identify based on the study’s eligibility criteria. The primary author, a nurse experienced in the care of YMC, assisted participants in determining eligibility if needed, and eligibility was further confirmed through the stories that were shared. However, it is possible that participants could have self-identified incorrectly.

Another limitation is the difficulty we faced in recruiting diverse participants. While we ensured that we explored a variety of contexts through maximum variation sampling, we recognize that selection bias inevitably limited the diversity of experiences that were explored. Some families declined to participate, as they were disillusioned by previous experiences with research or felt too traumatized or overwhelmed by ongoing experiences with transition. Therefore, this study may not have captured the experiences of those who struggle most with transition.

## 6 Conclusions

In summary, families of YMC are forced to adapt to an imposed transition, and they do so through a process of survival, sacrifice, persistence, and reframing. Their reflections lend valuable insight into the inequities and inefficiencies of the health care, social, and education systems. Future strategies should optimize families’ natural adaptation processes by drawing on their strengths and supporting areas of greatest need. Like the families in this study—who used creativity and resourcefulness to pursue a good life for their youth—clinicians, researchers, and decision makers need to work together to establish innovative approaches to care that push the bounds of our outdated and resource-limited systems.

## Funding

This work was supported by the Hospital for Sick Children Foundation, Norman Saunders Complex Care Initiative.

## Ethical Statement

This study received ethical approval from the Hamilton Integrated Research Ethics Board under project number #11184. Informed consent was obtained from all participants electronically and was reaffirmed verbally at the start of each interview.

## CRediT authorship contribution statement

**Lin Li:** Conceptualization, Data curation, Formal analysis, Funding acquisition, Investigation, Methodology, Project administration, Writing – original draft, Writing – review & editing. **Nancy Carter:** Conceptualization, Funding acquisition, Methodology, Supervision, Writing – review & editing. **Jan Willem Gorter:** Conceptualization, Funding acquisition, Methodology, Supervision, Writing – review & editing. **Linda Till:** Funding acquisition, Methodology, Validation, Writing – review & editing. **Marcy White:** Funding acquisition, Methodology, Validation, Writing – review & editing. **Patricia H Strachan:** Conceptualization, Formal analysis, Funding acquisition, Methodology, Supervision, Writing – review & editing.

## Declaration of Competing Interest

The authors declare that they have no known competing financial interests or personal relationships that could have appeared to influence the work reported in this paper.

## Data Availability

The data that has been used is confidential.
